# Optimisation of the DNA dipstick as a rapid extraction method for *Schistosoma japonicum* in infected mice samples and spiked human clinical samples

**DOI:** 10.1186/s40249-023-01118-8

**Published:** 2023-08-07

**Authors:** Oyime P. Aula, Donald P. McManus, Malcolm K. Jones, Hong You, Pengfei Cai, Catherine A. Gordon

**Affiliations:** 1 Molecular Parasitology Laboratory, QIMR Berghofer Medical Research Institute, Brisbane, Australia; 2 School of Public Health, Faculty of Medicine, The University of Queensland, Brisbane, Australia; 3 School of Veterinary Science, The University of Queensland, Brisbane, Queensland Australia; 4 Faculty of Medicine, The University of Queensland, Brisbane, Australia

**Keywords:** Schistosomiasis, *Schistosoma japonicum*, DNA dipstick, Loop-mediated isothermal amplification assay, Flocculation

## Abstract

**Background:**

Schistosomiasis remains a public health issue and the need for accurate and affordable diagnostics is crucial in the elimination of the disease. While molecular diagnostics are highly effective, they are expensive, with the main costs been associated with DNA extraction. The DNA dipstick is a rapid, affordable and simple purification method that allows DNA to be extracted from diagnostic samples within 30 s. We aimed to optimise the DNA dipstick method for samples from mice and egg-spiked human samples.

**Methods:**

Urine, blood and faeces were collected from mice exposed to *Schistosoma japonicum* infection at weekly intervals from Day 0 to Day 42. Urine and faecal samples were also collected from volunteer, uninfected humans and spiked with *S. japonicum* eggs. All samples were subject to several optimisation procedures and DNA extracted with the DNA dipstick. Amplification of the target DNA was carried out using LAMP and visualised using agarose gel electrophoresis and flocculation.

**Results:**

The DNA dipstick successfully identified *S. japonicum* from infected mice and human clinical samples spiked with cracked eggs or genomic DNA from *S. japonicum*. Amplification was observed from week 4 post infection in infected mice. For human samples, amplification was observed in sieved faecal samples, filtered urine samples heated at 95 °C for 30 min, and sera samples heated at 95 °C for 30 min.

**Conclusions:**

The DNA dipstick combined with LAMP has huge potential in providing cost-effective, simple and accurate detection of schistosomiasis infection in endemic regions. This will allow for rapid treatment, tracking outbreaks—such as occur after typhoons, leading to better health outcomes and contributing to control and eventual elimination of schistosomiasis.

## Background

Schistosomiasis is a neglected tropical disease (NTD) caused by infection with flukes from the genus *Schistosoma,* and is responsible for more than 200 million infections globally, with most of the infections concentrated in sub-Saharan Africa [1]. The majority of infections are reported in school-aged children from high proportion of studies aimed at this population. The disease is causes physical and cognitive impairment [2, 3]. Up to 20 million people in these low-income areas suffer severe chronic health consequences of the disease [4]. As of 2019, the global disease burden of schistosomiasis was 1.64 million disability-adjusted life years (DALYs) [5]. Human schistosomiasis is caused by three main species: *Schistosoma japonicum*, *S. mansoni* and *S. haematobium*.

The World Health Organization (WHO) has released a new road map for controlling or eliminating NTDs 2021–2030 [6], highlighting diagnostics as a major focus. Accurate, affordable and field-friendly diagnostics/surveillance tools are vital but missing components in control and elimination of schistosomiasis [6]. There are many lab-based diagnostics used in research for diagnosis of schistosomiasis, however few are standardised for clinical diagnostics and there is only one commercially available point-of-care (POC) diagnostic, the schistosomiasis POC-CCA cassette-based test, which utilises urine for diagnosis of *S. mansoni*, and with less sensitivity, *S. haematobium* [7, 8]. However, recent studies have identified high levels of false positives in patients tested from non-endemic areas which reduces the usefulness of this diagnostic [9].

Molecular diagnostics have shown to be highly sensitive and specific and have successfully been used in the detection of a wide range of parasites [10–14]. The starting point for most molecular applications begins with DNA extraction, which is usually the most expensive step in molecular diagnostics and is time consuming, requires sophisticated and expensive equipment, and trained personnel [15].

The DNA dipstick is an inexpensive, generic, rapid and simple cellulose-based DNA binding tool that has the ability to purify nucleic acids in less than 30 s and does not require specialised equipment including pipettes [16, 17]. The dipsticks are made from Whatman No. 1 filter paper that is coated with molten paraplast to create a hydrophobic handle, leaving an 8 mm unwaxed section for DNA binding [16, 17]. The DNA dipstick can then be used in conjunction with isothermal amplification, such as the loop mediated amplification assay (LAMP) [18]. Amplification does not require a thermocycler and can be carried out using a water bath or heat block. The amplification products can be visualised in a number of ways, such as turbidity, resulting from the formation of an insoluble white precipitate, colour change, via agarose gel electrophoresis or flocculation [17]. Flocculation utilises inexpensive low-density particles, activated charcoal and diatomaceous earth, which bind to amplified DNA and clump at the bottom of a tube, indicating a positive reaction. In the case of no amplification, the tube remains opaque [17]. Furthermore, one of the components of the flocculation solution, spermine, neutralises negative charges on DNA and other molecules in the flocculation solution, making it less sensitive to contaminants that may have been introduced from sample lysates [17, 19].

The DNA dipstick, when combined with LAMP, is a rapid tool with potential for POC testing in endemic regions with low socio-economic status that lack health infrastructure. We previously reported the use of this technique to detect DNA for schistosome eggs and crushed snails as a proof-of-concept [20]. Here, we describe the optimisation of the DNA dipstick and LAMP for the detection of *S. japonicum* in mice and human samples.

## Materials and methods

### Ethics statement

This study was approved by the QIMR Berghofer Medical Research Institute (QIMRB) Animal Ethics Committee (P288, P3706). This study was performed in accordance with the recommendations of the Australian code of practice for the care and use of animals for scientific purposes, 2004. This study was also approved by the QIMR Berghofer Human Ethics Committee and the Ethics Committee of the Research Institute for Tropical Medicine (RITM), Manila. Informed written consent was received from all study participants [21].

### Mouse *Schistosoma japonicum* infections and clinical sample collection

*Schistosoma japonicum* cercariae were shed from infected *Oncomelania hupensis hupensis* from China (Guichi County, Anhui) [Chinese strain] and *Oncomelania hupensis quadrasi* snails obtained from the Philippines (Irosin, near Sorsogon, Southern Luzon) [Philippines strain] [22].

**Fig. 1:**
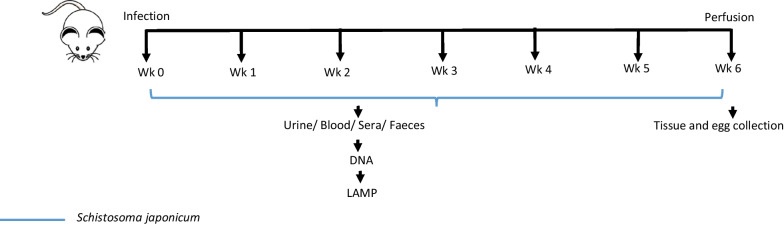
Outline for time point experiments in *S. japonicum* infected mice. Urine, sera, and faeces were collected from week 0, pre-infection, to week 6 when animals were perfused

Two groups of five female ARC Swiss mice were infected percutaneously with 70 *S. japonicum* cercariae; one group infected with Chinese strain *S. japonicum*, and the other with the Philippines strain. Samples were collected for 6 weeks from pre-infection (Week 0) to perfusion (Fig. 1). Blood samples were collected from five mice by tail vein puncture, allowed to clot for 30 min at room temperature, and serum separated by centrifuging at 2000 *g* for 10 min. The serum was carefully collected and transferred to a new tube. Both serum and the remaining clot were stored at − 80 °C. Urine samples were collected from all 5 mice and pooled together due to low volumes and stored at − 80 °C. Mice were placed on grid cages overnight and faecal samples collected and pooled together at each time point. The faecal samples were fixed in 80% ethanol and stored at 4 °C until the DNA dipstick extraction was carried out.

Parasite material in the form of eggs and adult worms were sourced from mice 6 weeks post infection (pi) after perfusion (Fig. 1). Eggs were isolated from the livers of infected mice and isolated by digestion using collagenase B as previously described [23].

### DNA dipstick extraction

The dipsticks were made from Whatman filter paper number 1 as previously described [16]. The filter paper was then cut into strips 44 mm long and 2 mm wide, leaving a small 4 mm^2^ unwaxed section for DNA binding to occur.

**Fig. 2:**
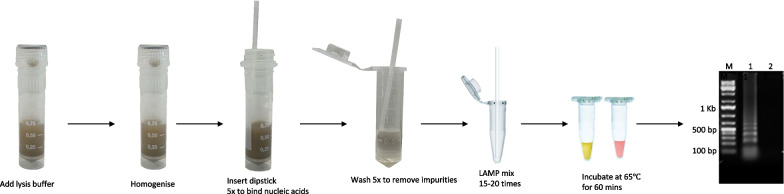
General workflow for DNA dipstick extraction. Modifications and optimisation steps varied depending on clinical sample used. The basic workflow involved incubation at 95 °C followed by homogenisation. After which, the DNA dipstick was dipped into the homogenate, dipped into wash buffer, then dipped into the LAMP reaction, and the LAMP reaction run. Results were viewed by colour change, and run on an agarose gel  to confirm results. LAMP: Loop Mediated Isothermal Amplification Assay

In order to purify DNA using the dipsticks, 200 µl lysis buffer [20 mmol/l Tris (pH 8), 25 mmol/l NaCl, and 2.5 mmol/l EDTA, 0.05%SDS] was added to the clinical samples and mixed before the DNA dipstick was dipped into the lysate five times. The dipstick was then dipped into 500 µl wash buffer [10 mmol/l Tris (pH 8.0), 0.1% Tween-20] five times and into the amplification mix 15–20 times (Fig. 2).

### LAMP optimisation

The primers targeting the 28S rDNA (Sj28S) region were utilised for *S. japonicum* [24]. The inner primers were tested with and without a TTTT spacer. The sequence for the primers with the spacers is as follows: FIP contained F1c, a TTTT spacer and the sequence (F2) complementary to F2c. BIP contained the sequence (B1c) complementary to B1, a TTTT spacer and B2. The outer primers were made up of the F3 and B3 sequences respectively. Spacer regions in FIP and BIP are in bold below.

Sj28S:

F3-GCTTTGTCCTTCGGGCATTA.

B3-GGTTTCGTAACGCCCAATGA.

FIP-ACGCAACTGCCAACGTGACATATTTTCTGGTCGGCTTGTTACTAGC.

BIP-TGGTAGACGATCCACCTGACCCTTTTCTCGCGCACATGTTAAACTC.

The reaction was carried out in a 25 µl mixture containing 12.5 µl WarmStart Colorimetric LAMP 2 × Master Mix (New England Biolabs, Ipswich, MA, USA), 1.6 µmol/l of each inner primer and 0.2 µmol/l of each outer primer (Integrated DNA Technologies, Melbourne, Australia). The dipstick was dipped into the master mix 3–15 times while 1 μl template DNA was used as positive control and 1 μl milliQ water was used as negative control. Reaction tubes were incubated at different temperatures and times: 63 °C or 65 °C for 30–60 min in a heat block, 63 °C or 65 °C for 30–60 min in a water bath, 63 °C or 65 °C for 60 min in a thermocycler, 63 °C or 65 °C for 90 min in a thermocycler and a third reaction at 65 ℃ for 60 min, followed by 5 min incubation at 80 °C to terminate the reaction.

### Analysis of amplification products

Positive LAMP results were visualised by a colour change from pink to yellow while negative products remained pink. For each reaction, 5 µl of the LAMP products were run on 2% agarose gel [1 × Tris–acetate-EDTA (TAE)], and a further 10 µl of the LAMP products was added to 30 µl flocculation solution.

### Flocculation assay

The flocculation solution contained 400 mg activated charcoal, 600 mg diatomaceous earth, 50 mmol/l Tris (ph 8), 10 mmol/l spermine and 1% (v/v) PEG 8000, all combined in a 50 ml solution. 30 µl of the flocculation solution was added to 10 µl of the amplification product. The tubes were gently flicked for about 10 s and stood in an upright position for 1 min. Positive reactions were observed when flocculation material clumped together with amplified DNA and settled at the bottom of the tube, while negative results remained dark and opaque.

### Positive controls

Genomic DNA from *S. japonicum* was extracted from eggs using the PrepEase DNA isolation kit (Affymetrix, Santa Clara, USA) according to the manufacturer’s guidelines. DNA concentration was quantified using the Nanodrop 1000 spectrophotometer (Thermo Scientific, Waltham, USA) and used as positive control.

Eggs of *S. japonicum* were isolated from the liver of infected mice as previously described [23]. An aliquot of 50 µl from the resultant isolated egg suspension was then added to 50 µl of lysis buffer and then cracked using a handheld mortar and pestle (Kimble, Vineland, NJ, USA) as previously described [20]. The homogenised material was then kept at − 20 °C until required for use as a positive control in the LAMP experiments.

When used for the positive control samples, the DNA dipstick was dipped into the homogenised eggs samples five times, dipped in wash buffer five times and into the LAMP reaction tube 15–20 times.

### DNA dipstick optimisation for extracting parasite DNA from mice samples

The first step to optimising the DNA dipstick in mice was to test if the dipsticks had the ability to capture DNA from clinical samples, without causing inhibition of the LAMP reaction with any carry over from mice samples. Negative urine, sera and faecal samples were spiked with 2 µl *S. japonicum* genomic DNA and dipstick performed. Next, urine, blood and faecal samples were collected from experimentally infected *S. japonicum* mice and DNA dipstick in conjunction with LAMP was carried out as described in the sections below.

#### Urine

##### Seeding experiments

Urine from uninfected mice were divided into nine aliquots of 500 µl each in 1.7 ml Eppendorf tubes and seeded with cracked *S. japonicum* eggs. 500 µl of lysis buffer was added to all tubes and then subjected to three different incubation temperatures (37 °C, 65 °C and 95 °C) and three different incubation times (10, 20, and 30 min) to determine the optimal temperature and time for cell lysis to occur (Table 1).

**Table 1:** Permutations for mice samples

	Faeces	Urine	Sera
DNA spiking	✓	✓	✓
Heat	✓	✓	✓
PK at RT	✓	✓	✓
Vortex	✓	✓	✓
Heat + PK	✓	✓	✓
Heat + PK + vortex	✓	✓	✓
PK + vortex	✓	✓	✓
Heat + vortex	✓	✓	✓
Heat + hand-held mortar and pestle	✓	✓	✓
Lysis buffer mix	✓	✓	✓
FTA card + lysis buffer	✓	✓	✓
FTA card + water	✓	✓	✓
FTA card + heat (50 °C)	✓	✓	✓
FTA card + heat (50 °C) for 30 min	✓	✓	✓
FTA card + heat (95 °C) for 30 min	✓	✓	✓
Sample + dipstick	✓	✓	✓
Egg spiking at RT	✓	✓	✓
Egg spiking at RT for 30 min	✓	✓	✓
Egg spiking at 4 °C O/N	✓	✓	✓
Filtration		✓	×
Dipstick + WB + Tween 20	×	✓	✓
Dipstick + WB − Tween 20	×	✓	✓

*PK Proteinase K, *RT* room temperature, *O/N* overnight, *FTA* flinders Technology Associates, *WB* wash buffer

##### Infected mouse urine

Urine samples were collected from five infected mice starting at week 0, prior to infection, and then every week until week 6 post infection (pi) (Fig. 1). Urine collected from mice were pooled together in 1.5 µl Eppendorf tubes. From the pooled urine, two aliquots of 50 µl were pipetted into separate tubes labelled A and B. 50 µl lysis buffer was added to each tube. Tube A was incubated at 95 °C for 30 min while Tube B was incubated at room temperature for 30 min. DNA was extracted from both groups using the dipstick as described above (Fig. 2) and inserted into the LAMP reaction. The reaction was incubated at 65 °C for 40 min in a water bath or 90 min in a thermocycler.

#### Faeces

Mouse faecal pellets were collected from five mice from week 0, prior to infection, and then every week up to week 6 pi. Six pellets from the pooled mice faeces were placed into three separate tubes and 300 µl of lysis buffer was added to each. Each tube was then homogenised and passed through either: (1) 250 µm mesh, (2) 250 µm and 100 µm mesh, or (3) 250 µm and 40 µm (pore opening size). For (1) and (2), flow-through was collected and concentrated by centrifuging at 10,000 *g* for 5 min. For group (3), the residue on the 40 µm was rinsed into a 50 ml and concentrated again by centrifugation. The DNA dipstick was performed as previously described and inserted into the LAMP reaction (Fig. 2). The reaction was incubated at 65 °C for 40 min in a water bath or 90 min in a thermocycler.

#### Blood

Blood was collected from five mice from week 0, and then every week up to week 6 pi, and placed into 1.5 ml Eppendorf tubes. Blood was left to clot for 30 min before centrifuging at 800 *g* for ten minutes to separate the whole blood and serum. Serum was removed and placed into a new 1.5 ml Eppendorf tube. From each mouse, 50 µl of blood and 50 µl serum were placed into new tubes and 50 µl of lysis buffer added to each. The tubes were vortexed briefly and then each tube was divided into two tubes. Tube one was heated to 95 °C for 30 min while Tube two was incubated at room temperature for 30 min. DNA dipstick was then performed and added to the LAMP reaction. Colour change was examined once the LAMP reaction was completed and then gel electrophoresis and flocculation were performed.

All testing conditions trialled for mice clinical samples can be seen in Table 1.

### Dipstick for clinical samples from humans

Urine and faecal samples from uninfected humans were acquired from laboratory volunteers and tested using qPCR for *S. japonicum* infection described in Gordon et al*.* [25] before use in this study.

#### Seeding experiments for optimisation of method in human clinical samples

##### Urine

Negative urine was divided into 5 ml aliquots in a 15 ml tube. Tubes were: (1) spiked with 10 µl genomic *S. japonicum* DNA or (2) seeded with approximately 200 eggs and DNA dipstick performed. To test the effect of filtration, further 15 ml tubes containing 5 ml of negative human urine was either (1) spiked with genomic DNA or (2) cracked eggs, filtered through a Whatman filter paper 2 and left to dry overnight. A Harris Uni-Core 3.0 mm hole punch (Interpath Services/GE/Whatman) was used to remove 3.0 mm disks of each sample into 1.5 ml Eppendorf tubes, after which 200 µl lysis buffer was added to the tubes containing the filter disks and heated at 95 °C for 30 min. The DNA Dipstick and LAMP were then performed.

In a separate tube, 10 ml of negative human urine was seeded with approximately 1000 eggs and tested under different temperatures and times as shown in Table 2.

**Table 2:** Permutations for human clinical samples

	Faeces	Urine	Sera
1	Heat + metal beads	Genomic DNA	Naturally infected samples
2	Metal beads—shake vigorously	Cracked eggs	Naturally infected samples + heat
3	Metal beads + PK	Genomic DNA + filter paper	–
4	Metal beads + PK + heat	Cracked eggs + filter paper	–
5	Silica beads + PK	Cracked eggs + 37 °C + 10 min	–
6	Silica beads + heat	Cracked eggs + 65 °C + 10 min	–
7	Silica beads + PK + heat	Cracked eggs + 95 °C + 10 min	–
8	–	Cracked eggs + 37 °C + 20 min	–
9	–	Cracked eggs + 65 °C + 20 min	–
10	–	Cracked eggs + 95 °C + 20 min	–
11	–	Cracked eggs + 37 °C + 30 min	–
12	–	Cracked eggs + 65 °C + 30 min	–
13	–	Cracked eggs + 95 °C + 30 min	–
14	–	Naturally infected samples filtered and allowed to dry O/N + lysis buffer	–
15	–	Naturally infected samples filtered and allowed to dry O/N + 600 µl milliQ water	–

*Lysis buffer was added in all permutations except samples where genomic DNA was used

*PK* Proteinase K, *O/N* overnight, – not applicable

##### Faeces

**Fig. 3:**
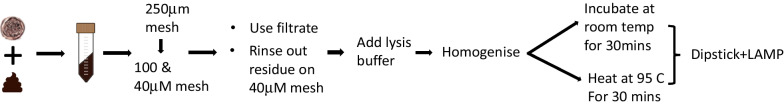
Workflow for human stool samples seeded with cracked *Shistosoma japonicum* eggs. Eggs were added to negative faeces and passed through a 250 µm (pore opening size) sieve and then either sieved through a 100 µm mesh, and the filtrate retained, or on to a 40 µm sieve and the residue washed into a new tube. The resulting filtrates and residue were divided into two groups, and incubated at room temperature or at 95 °C for 30 min. DNA dipstick and LAMP were then performed. LAMP: Loop Mediated Isothermal Amplification Assay

The DNA dipstick was tested on negative human faecal samples by spiking negative faecal samples with *S. japonicum* eggs isolated from mice livers. Three groups were prepared with moderate infections (100–399 eggs per gram of faeces [EPG]) by adding approximately 1000 eggs to 5 g of human faeces and re-suspended in 30 ml milliQ water and each group subjected to different conditions (Table 2):

1. The faecal sample was incubated at room temperature for 30 min and centrifuged at 13,000 *g* for 5 min and supernatant removed. The sediment was re-suspended in 1.5 ml lysis buffer and homogenised using a battery operated mortar and pestle (Kimble, USA) for 3 rounds of 40 secs, following which the DNA dipstick and LAMP were then performed.
2. 1 ml of milliQ water was added to 5 g faeces seeded with 1000 eggs in a 10 ml tube. 1 ml from the mix was transferred to a 1.7 ml Eppendorf tube and homogenised using a handheld mortar and pestle for 5 min and vortexed for 1 min. The solution was left to stand for 2–5 min before DNA dipstick was performed. The faecal matter was filtered through a 250 μm mesh to get rid of particles that could inhibit DNA extraction, and the DNA dipstick and LAMP performed.
3. The faecal sample was passed through a 250 μm mesh and then either through a 100 or 40 μm mesh. The filtrate from the 100 μm mesh was collected into a beaker while the residue from the 40 μm mesh was rinsed into a beaker (Fig. 3). Lysis buffer was added to the 100 μm filtrate or 40 μm residue, homogenised and incubated. Dipstick and LAMP were then performed.

In all faecal sample trials, the resultant homogenates were divided into two groups: Group 1 was incubated at room temperature while Group 2 was heated at 95 °C for 30 min (Fig. 3).

### Naturally infected human clinical samples

Naturally infected human urine and sera clinical samples were originally collected in 2015 from an *S. japonicum* endemic area of the Philippines and stored at − 80 °C. The infection status with *S. japonicum* had previously been determined by digital droplet PCR and Kato–Katz (KK) [21].

#### Urine

Naturally infected human urine samples were thawed and divided into 5 ml aliquots for optimisation experiments. Two groups were created with 5 ml urine samples placed into 15 ml tubes and centrifuged at 4000 *g* for 5 min. Urine samples were then subjected to the following conditions:

1. The supernatant was removed and the residue re-suspended in 300 µl lysis buffer and vortexed for 5 s. The DNA dipstick, followed by LAMP was then performed.
2. Another 5 ml of infected human urine was filtered through a Whatman filter paper 2 and left overnight to dry. A Harris Uni-Core 3.0 mm whole punch (Interpath Services/GE/Whatman) was used to remove 3.0 mm disks of each sample into 1.5 ml Eppendorf tubes. 200 µl lysis buffer was added to the disks and heated at 95 ℃ for 30 min. The DNA dipstick, followed by LAMP were then performed.

#### Sera

Three naturally-infected samples that had previously tested positive for *S. japonicum* by ddPCR [21] were thawed. 50 µl lysis buffer was added to 50 µl sera and heated at 95 °C for 30 min for DNA lysis to occur. This was followed by DNA dipstick and LAMP.

## Results

The optimal amplification results were obtained for mice samples when the reaction was incubated at 65 °C for 40 min in a water bath or 65 °C for 90 min in a thermocycler. Similarly, optimal amplification results were observed for seeded human faecal and urine samples, as well as naturally infected sera samples, when the reaction was incubated at 65 °C for 90 min. Successful amplification was viewed by either colour change from pink to yellow, DNA like bands on a 2% agarose gel, or adding flocculation solution after LAMP reactions had finished. The flocculation results matched results obtained from the agarose gel electrophoresis.

### DNA dipstick optimisation for extracting parasite DNA from clinical samples from mice

#### Seeded urine and sera samples

**Fig. 4:**
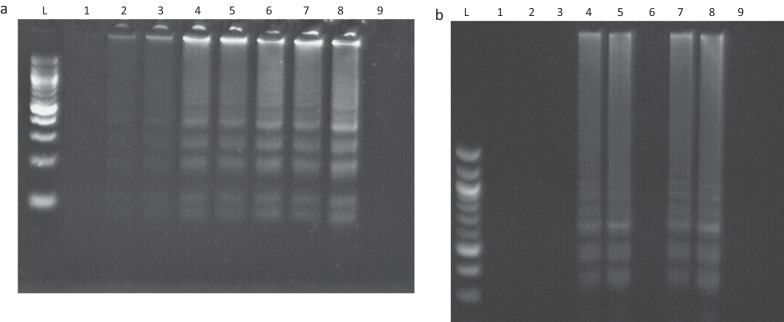
Optimisation of the DNA dipstick on spiked mice urine and sera.** a** DNA spiking of negative mice urine and sera. Amplification was observed after heating at 65 °C for 40 min in a water bath or 65 °C for 90 min in a thermocycler. L: Ladder, 1–3: Urine, 4–7: Sera; 8: Positive control, 9: NTC control. **b** *Schistosoma japonicum* DNA spiked mouse urine with different pre amplification conditions. 1: PK, 2: Heat, 3: Vortex, 4: PK + heat + vortex, 5: PK + heat, 6: PK + vortex, 7: Heat + vortex, 8: Positive control, 9: NTC control. PK: Proteinase K, heat: 95 °C

Urine and sera samples from mice spiked with 2 μl of genomic *S. japonicum* DNA was successfully extracted using the DNA dipstick and amplified by LAMP (Fig. 4a). Sera and urine samples incubated at 95 °C for 30 min also showed stronger band intensity compared to samples incubated at room temperature for 30 min.

Negative human urine and sera samples were also seeded with cracked eggs and incubated at room temperature or 95 °C for 30 min, following which successful amplification was observed for both heated urine and sera samples (Fig. 4).

#### Seeded faecal samples

Mouse faeces spiked with 2 µl of *S. japonicum* DNA did not show successful amplification (data not shown) after extraction with the DNA dipstick and subsequent LAMP assay. Thus, to assess whether inhibitors present in faeces and the presence of bulk debris prevented amplification of parasite DNA, faeces was sieved through different sized meshes (250 µm, 100 µm, and 40 µm pore sizes) and genomic DNA added to the sieved faecal material, which successfully showed amplification after extraction with the DNA dipstick and LAMP.

### Experimentally infected mice samples

**Fig. 5:**
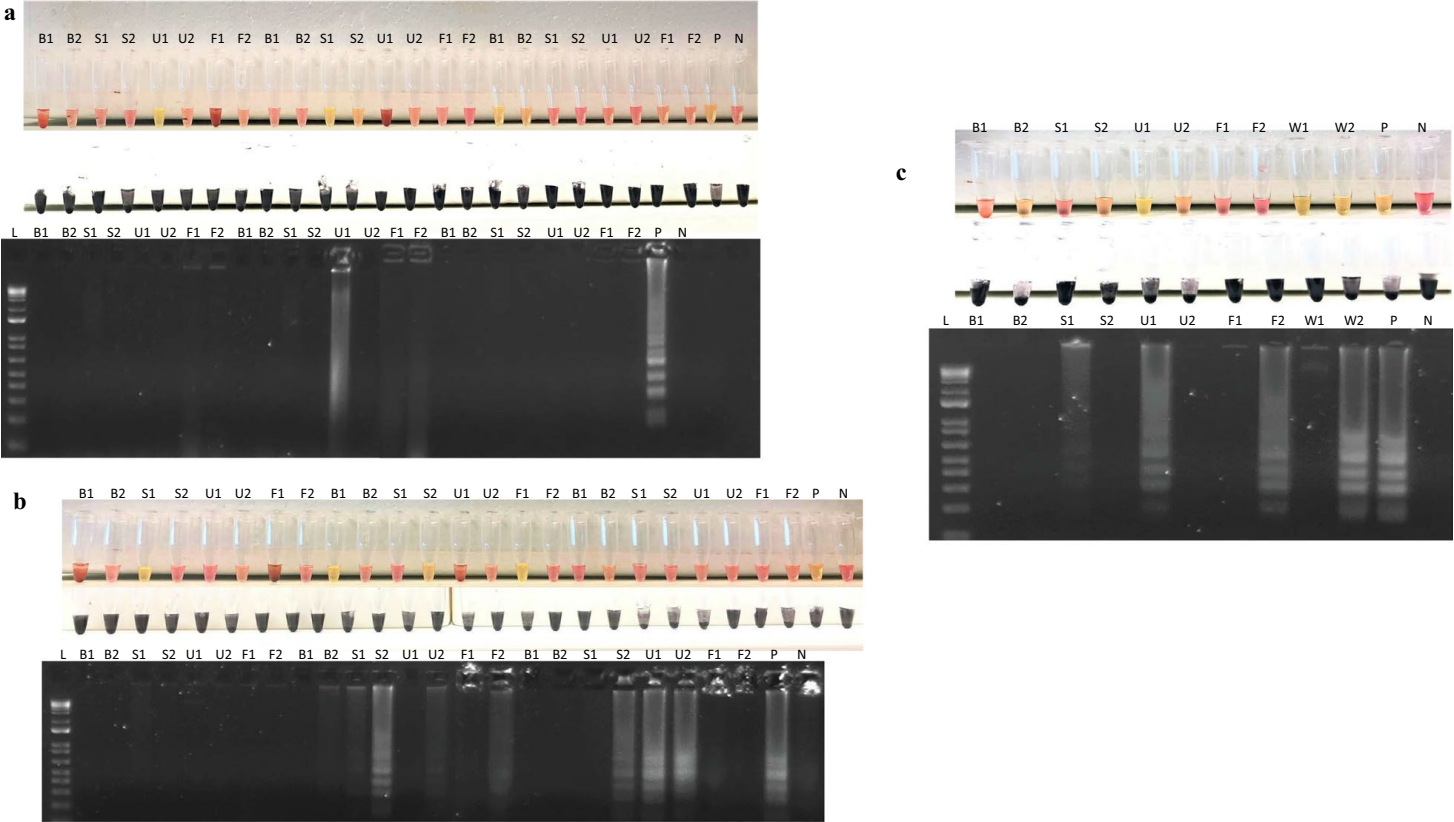
**a**–**c** Dipstick and LAMP assay for clinical samples from mice infected with *Schistosoma japonicum* from week 0 to week 6, visualised by colour change after incubation (top), flocculation assay (middle) or agarose gel electrophoresis (bottom). B1: Unheated blood; B2: Heated blood; S1: Unheated sera; S1: Heated sera; U1: Unheated urine; U1: Heated urine; F1: Unheated faeces; F1: Heated faeces; W1: Unheated *S. japonicum* worms; W2: Heated *S. japonicum* worms; P: Positive control; N: Negative control

Following the successful amplification of the seeded samples, the DNA dipstick was performed on experimentally infected mice samples from 0 to 6 weeks. Amplification was observed from week 4 pi in both heated and unheated urine, and sera samples, and at week 6 pi for faeces (Fig. 5).

### Seeding experiments for optimisation of method in human clinical samples (urine and faeces)

**Fig. 6:**
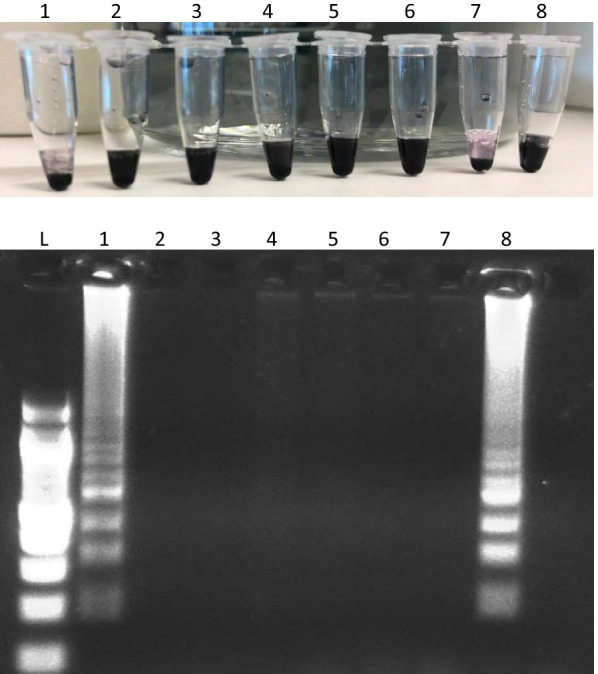
Optimisation of the dipstick on naturally infected human faecal samples sieved through a 40 µm or 100 µm mesh. Samples had a combination of either proteinase K (PK) added, heating (95 °C), or both prior to DNA extraction using the DNA dipstick and amplification via the LAMP assay. Amplification DNA was visualised by flocculation assay (top) or agarose gel electrophoresis (bottom). 1: 40 µm + heat; 2: 40 µm + PK + heat; 3: 40 µm + PK; 4: 40 µm; 5: 100 µm + heat; 6: 100 µm + PK + heat; 7: 100 µm + PK; 8: Positive control; 9: Negative control

To assess the effectiveness of the DNA dipstick, and to test for potential inhibition occurring in either sample type, 2 µl of genomic DNA was added to negative human urine and faeces and then DNA extracted with the DNA dipstick, followed by LAMP. Amplification was not observed in either clinical sample. Urine and faeces were then seeded with cracked eggs as described above. Amplification was observed in the faecal residue that had been sieved (through a 250 µm mesh and on to a 40 µm mesh) and heated at 95 °C for 30 min (Fig. 6).

### Naturally infected human clinical samples

#### Urine

Amplification was not observed in unheated naturally infected urine samples. However, amplification was observed in urine samples filtered through the Whatman filter paper and incubated at 95 °C for 30 min (not shown).

#### Sera

**Fig. 7:**
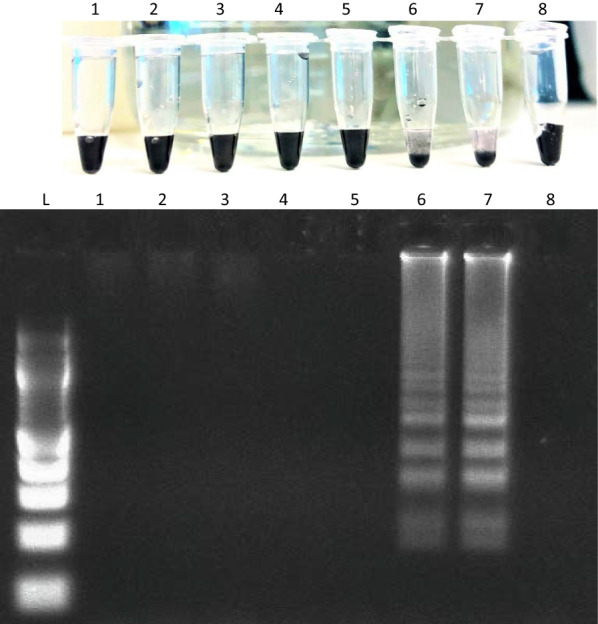
Optimisation of the dipstick on naturally infected human sera samples. 1, 2, 3: Unheated sera; 4, 5, 6: Heated at 95 °C for 30 min; 7: Positive control; 8: Negative control

Amplification was not observed in unheated naturally infected sera samples. Amplification was however observed in one of three sera samples after incubation at 95 °C for 30 min (Fig. 7).

## Discussion

One of the WHO 2030 goals is to eliminate schistosomiasis as a public health issue. Although treatment of the disease with the use of praziquantel plays a role in the control of the disease, resistance to the drug after mass drug administration (MDA) is a concern [26]. Regular MDA also reduces the infection intensity below the level of which Kato–Katz—traditionally used to determine infection status in endemic areas, can routinely detect. Hence, more sensitive and accurate diagnostics to detect infection at acute and chronic stages for targeted treatment are necessary. While the current molecular diagnostics are highly accurate, the need for sophisticated equipment and skilled personnel limit their application in resource-poor settings.

The development of POC diagnostics are a major part of the World Health Organisation’s (WHO) new 2021–2030 roadmap for NTD control. For a diagnostic method to be classified as a POC test, it must satisfy the ASSURED (Affordable, Sensitive, Specific, User-friendly, Rapid and Robust, Equipment-free, Deliverable) criteria set out by the WHO. These criteria have recently been revised to REASSURED to include Real-time connectivity, Ease of specimen collection, and Environmental friendliness [27]. Current molecular diagnostics require expensive kits and chemicals as well as laboratory equipment with the cost of DNA extraction making up the main cost of molecular diagnosis. Hence, the DNA dipstick can provide a rapid and affordable approach for nucleic acid purification and a tool for improved parasite diagnosis at the POC [16, 20].

This study aimed to optimise the DNA dipstick in conjunction with LAMP as a potential POC testing tool using a mouse model infected with *S. japonicum*. The DNA dipstick presents a novel method for DNA purification from parasite infected samples at low cost compared to traditional DNA extraction methods. The results demonstrated that *S. japonicum* DNA could be detected from 4 weeks pi in mouse sera and urine, and week 6 pi for faecal samples. In sera and urine, cell free DNA (cfDNA) is being detected as eggs are only released in the faeces in *S. japonicum* infections and only after 5–6 weeks. *S. japonicum* cfDNA was also detected one week pi in mice clinical samples (urine, sera, faeces) using ddPCR following kit based DNA extraction in previous studies [28]. DNA was not detected in mouse faecal samples until week 6 pi, likely due to the loss of cfDNA from the sieving stages. Additionally, DNA was only detected in the residue, due to the presence of eggs that had been trapped from sieving the sample.

### Seeded mouse samples

Trials initially began with seeding clinical mouse samples with *S. japonicum* DNA to test if inhibitors present in clinical samples would interfere with successful amplification post DNA extraction with the DNA dipstick. We successfully showed amplification in *S. japonicum* DNA seeded and *S. japonicum* cracked egg seeded mouse urine, sera, and faces, although faeces required sieving to remove bulk debris before successful amplification was observed.

### Seeded human clinical samples

Successful amplification was observed in both sample types when seeded with cracked eggs and heated, but no amplification was observed in the unheated samples. Amplification was also observed in DNA spiked urine samples but not observed in faecal samples that were seeded with genomic DNA, as the DNA would have been removed in the sieving steps. This has important implications for removal of cfDNA in naturally infected faecal samples, as pre-patent (pre-egg laying) detection using molecular methods requires presence of cfDNA. DNA and egg spiking were not carried out on negative sera as we were unable to obtain negative human sera samples.

### Naturally infected human clinical samples

In addition to the mouse samples, the DNA dipstick method was tested on a subset of infected human clinical samples (urine, sera) from a prior study conducted in 2015 in the Philippines [21]. Amplification was not observed for urine samples when the DNA dipstick was directly applied to the urine sample. Successful amplification was however observed after the urine samples were filtered to concentrate any *S. japonicum* DNA present, and when heated to 95 °C. Amplification was not observed in filtered but unheated samples. Amplification of infected human sera was also observed only in the heated samples.

### Visualisation methods

The LAMP reagent used was a colorimetric version that changes colour from pink to yellow when successful amplification occurred. The colour change is dependent on a pH change, so with increasing DNA, the reaction becomes more acidic, prompting the colour change. We observed colour changes occurring directly after the DNA dipstick was applied to the LAMP reaction for urine—an immediate change from pink to yellow. Although urine gets its yellow colour due to the presence of urobilin obtained from the breakdown of old red blood cells, the immediate colour change observed here was likely due to the acidity of urine. Therefore, alternative visualisation methods were investigated. DNA-like bands were observed when LAMP amplification products were run on agarose gel, however the use of agarose gel electrophoresis is not practical in field settings due to the high cost of the equipment needed, and the risk of cross-contamination. Flocculation was then investigated as a more field-friendly visualisation method. Both gel electrophoresis and flocculation were run for all clinical samples, and results were in perfect agreement for both methods, validating flocculation as a visualisation method for determining positive and negative results. The flocculation solution also has minimal cross-contamination risk due to the low pH used in the solution and the presence of a DNA compaction agent, spermine, in the flocculation solution. Spermine has the ability to neutralise the repelling negative charges on the DNA molecules by binding to the major and minor grooves of the DNA helix [19]. The presence of these two factors makes the flocculation solution less sensitive to contaminants that may have been introduced from sample lysates [17]. This makes the flocculation solution an ideal visual read-out that could be applied in field-settings.

Visualisation of reactions within tubes was a limitation due to immediate colour change with insertion of samples from mice, particularly urine, due to change in pH. However, flocculation was successfully used and gave the same results as per gel electrophoresis. Additionally, further optimisation of the DNA dipstick for different clinical samples utilising naturally infected human samples is required to further streamline the process and make it more field friendly.

## Conclusions

Our study demonstrated that DNA dipstick in conjunction with LAMP is a promising, potential POC for the diagnosis of schistosomiasis and other NTDs. Optimisation was successful in infected faecal, urine and sera samples obtained from mice, in heated seeded human faecal and urine samples and in naturally infected heated sera samples. Further research is needed for the evaluation of this technique in a wider range of human clinical samples to improve the assessment of its potential in field-settings, as well as investigating other methods to concentrate faecal samples without removing any cfDNA present. With its low-cost, rapidity and ease of use, the DNA dipstick has the potential for field diagnosis of schistosomiasis in endemic areas.

## Data Availability

The data and materials presented in this study are available in the article.

## Abbreviations

B3: Backward outer primer
BIP: Backward inner primer
cfDNA: Cell-free DNA
ddPCR: Droplet digital PCR
DALYs: Disability-adjusted life years
DNA: Deoxyribonucleic acid
EDTA: Ethylenediaminetetraacetic acid
F3: Forward outer primer
FIP: Forward inner primer
KK: Kato-Katz
LAMP: Loop Mediated Isothermal Amplification Assay
MDA: Mass Drug Administration
NTD: Neglected tropical disease
PCR: Polymerase chain reaction
pi: Post infection
POC: Point-of-care
POC-CCA: Point-of-care circulating cathodic antigen
QIMRB: QIMR Berghofer Medical Research Institute
RITM: Research Institute for Tropical Medicine
TAE: Tris-acetate-EDTA
WHO: World Health Organization
